# Gender Gap in the ERASMUS Mobility Program

**DOI:** 10.1371/journal.pone.0149514

**Published:** 2016-02-22

**Authors:** Lucas Böttcher, Nuno A. M. Araújo, Jan Nagler, José F. F. Mendes, Dirk Helbing, Hans J. Herrmann

**Affiliations:** 1 Computational Physics for Engineering Materials, Institute for Building Materials, ETH Zurich, Zurich, Switzerland; 2 Departamento de Física, Faculdade de Ciências and Centro de Física Teórica e Computacional, Universidade de Lisboa, Lisboa, Portugal; 3 Departamento de Física and I3N, Universidade de Aveiro, Aveiro, Portugal; 4 Professorship of Computational Social Science, ETH Zurich, Zurich, Switzerland; 5 Departamento de Física, Universidade Federal do Ceará, Fortaleza, Brazil; University of Maribor, SLOVENIA

## Abstract

Studying abroad has become very popular among students. The ERASMUS mobility program is one of the largest international student exchange programs in the world, which has supported already more than three million participants since 1987. We analyzed the mobility pattern within this program in 2011-12 and found a gender gap across countries and subject areas. Namely, for almost all participating countries, female students are over-represented in the ERASMUS program when compared to the entire population of tertiary students. The same tendency is observed across different subject areas. We also found a gender asymmetry in the geographical distribution of hosting institutions, with a bias of male students in Scandinavian countries. However, a detailed analysis reveals that this latter asymmetry is rather driven by subject and consistent with the distribution of gender ratios among subject areas.

## Introduction

Statistical analyses of big data sets have revealed interesting patterns related to human mobility. For example, from the trajectory of mobile phone users, it was possible to identify temporal and spacial regularity in the mobility patterns, with a characteristic travel distance and a small set of frequently visited locations for each individual [1]. Using data from global connectivity and epidemic spreading, Brockmann and Helbing could successfully predict the disease arrival time and/or sources for different diseases [2]. Also, from the database of airports and alternative connections between these airports it was possible to reveal a core-periphery structure in the World Airline Network, consisting of a strongly connected core and a weakly connected, tree-like, periphery [3]. Here, we use similar tools to evaluate possible gender differences in the mobility pattern of ERASMUS students.

ERASMUS is an European Unity exchange program that provides financial support to European students to study abroad. It brings together more than four thousand academic institutions and companies across 33 countries and aims at boosting the participants x2019; job prospects by encouraging international mobility and promoting the development of personal skills, such as intercultural awareness, openness, and flexibility [4–8]. The participation in the ERASMUS program has increased impressively, from a mere three thousand participants in 1987 to 252827 in 2012 [4, 6]. The number of participants in the 2011–12 edition corresponds to almost 1% of all tertiary students [9]. This impressive level of participation makes the ERASMUS program an excellent example to study the enrollment of students in exchange programs and to identify mobility patterns.

A comparative study of ERASMUS and non-ERASMUS students concludes that the decision to participate is mainly affected by professional aspects and personal preferences, although a financial barrier is also identified [10]. Studies of the network of ERASMUS institutions show that the choice for a country is positively correlated to its number of top ranked universities [11] and that students are typically biased towards institutions that were previously selected by their home-university fellows [12]. The personal motivation for participating in mobility programs should be interpreted in the context of the social environment, personal experiences, and the macroeconomic situation in the country of residence [13]. So far, however, gender differences have not been thoroughly studied. The number of female students in tertiary education is definitely on the rise [14]. According to the EUROSTAT tertiary education statistics, the number of female students in EU-28 countries even surpassed the number of their male fellows [9]. Are the mobility patterns of male and female students the same or different? Here we show that, in the ERASMUS program in 2011–12, female students are consistently over-represented, even when considering their majority in tertiary education. This result is in sharp contrast to the labor market, where empirical studies suggest that the mobility of female workers is lower than the one of their male counterparts [15].

## Materials and Methods

The ERASMUS student mobility data set for the 2011–12 edition contains the list of all participants and their home- and host-institution/country, gender, age, nationality and subject area. Home- and host-academic institutions are represented by their institution code that is uniquely defined. We have the list of codes and names for 4466 institutions. For 1915 of them, there is no information available about their official name and therefore we decided to remove them from the list. The resulting data set consists of 2551 universities and 199488 participants. We provide the entire data set as S1 File.

The data set also contains information about the type of mobility: mobility for study (between two academic institutions), industrial placement (between universities and industrial partners) or combinations of both. In the latter case we considered only the university as the host-institution. In the data, there are 204744 university exchanges, 48083 industrial placements and only 438 combined exchanges.

Additional statistical information about the ERASMUS program was obtained from the statistical reports of the European Union [6]. The tertiary education statistics in the ERASMUS countries was obtained from EUROSTAT [9].

To investigate the over-representation of female students, we first compared the tertiary education statistics with the ERASMUS data using a null model for which we assume that the population of ERASMUS students is randomly drawn from the student population of the participating countries. Both data sets are analyzed based on the comparison of proportions of students in different subject areas and countries. In order to better visualize the difference we also show the ratio of both values. Our analysis is mostly based on averaging over certain data set sub-populations.

## Results

In 2011–12, 153468 ERASMUS participants (about 61%) were female students. This percentage is even 1.13 times higher than the fraction of female students attending tertiary education in the ERASMUS countries. This higher rate of participation is practically the same for industrial internships and university exchanges. Note that, if participants were randomly drawn from the entire population of 24606715 tertiary students, the expected number of female participants (136527) would differ from the actually observed one (153468) more than 60 times the standard deviation (251). Thus, it is highly improbable that the over-representation of female students is a mere statistical fluctuation. Below we analyze this gender patterns across subject areas and countries.

**Fig 1:**
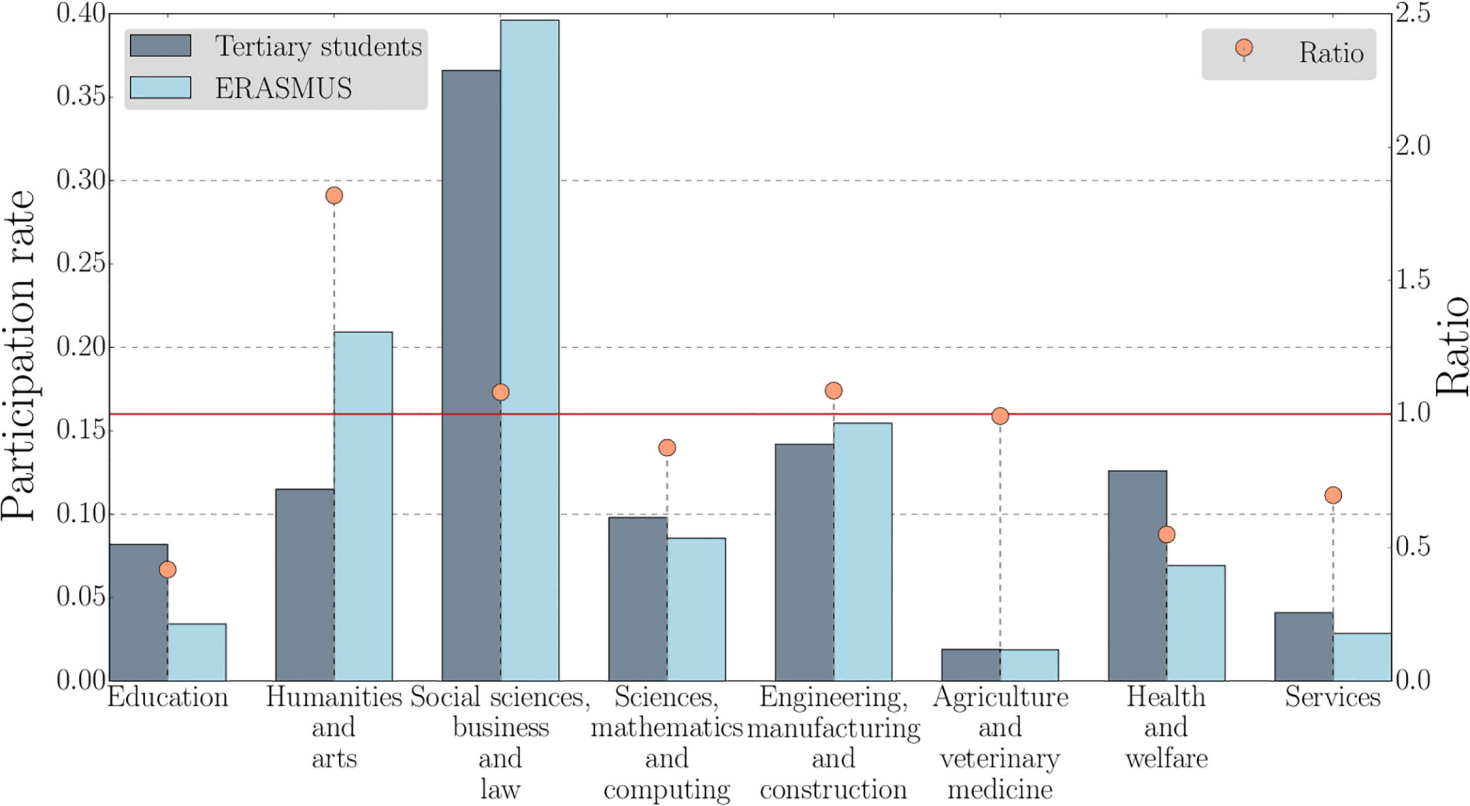
The participation rate in the ERASMUS program depends on the subject area. Participation rate in different subject areas in the 2011–12 ERASMUS program (light blue) and in tertiary education in the participating countries in 2011 (dark blue). The orange circles are the ratios of these fractions. While *humanities and arts*, *social sciences, business and law*, *engineering, manufacturing and construction* are over-represented in the ERASMUS program, the others are under-represented.

**Fig 2:**
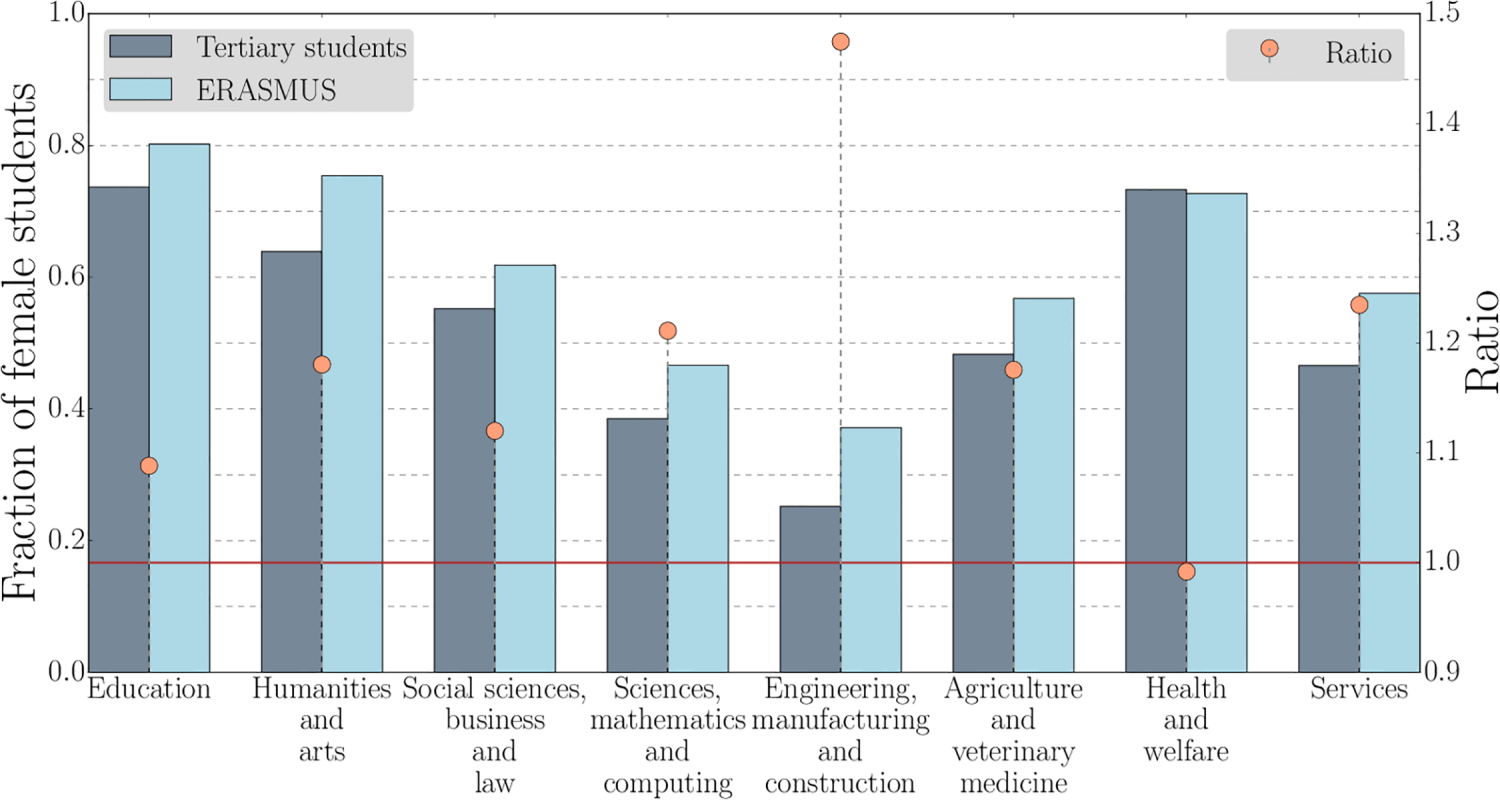
The over-representation of female students in the ERASMUS program is systematic across subject areas. Fraction of female students in different subject areas in the 2011–12 ERASMUS program (light blue) and in tertiary education in the participating countries in 2011 (dark blue). The orange circles are the ratios of these fractions. For almost all subject areas, female students are over-represented in the ERASMUS program. The only exception is *health and welfare*, where the ratio is balanced.

According to the *International Standard Classification of Education* (ISCED), the population of tertiary students is divided into eight subject areas: *education*; *humanities and arts*; *social sciences, business and law*; *sciences, mathematics and computing*; *engineering, manufacturing and construction*; *agriculture and veterinary medicine*; *health and welfare*; and *services*. Fig 1 shows the participation rate over these subject areas for the ERASMUS program and for the entire tertiary education population in the ERASMUS countries. One sees that certain subjects are clearly over-represented in the ERASMUS program. For example, *humanities and arts* rank second in terms of ERASMUS participants while they rank fourth in the total population of tertiary students. By contrast, the participation of *education* students is very low. Fig 2 shows the fractions of female students in tertiary education and ERASMUS together with the ratio between them. In line with the agglomerated data, females are over-represented in the ERASMUS program across all subject areas, except for *health and welfare*. For *engineering, manufacturing and construction*, an area typically dominated by male students, the ratio is almost 1.5 times higher.

**Fig 3:**
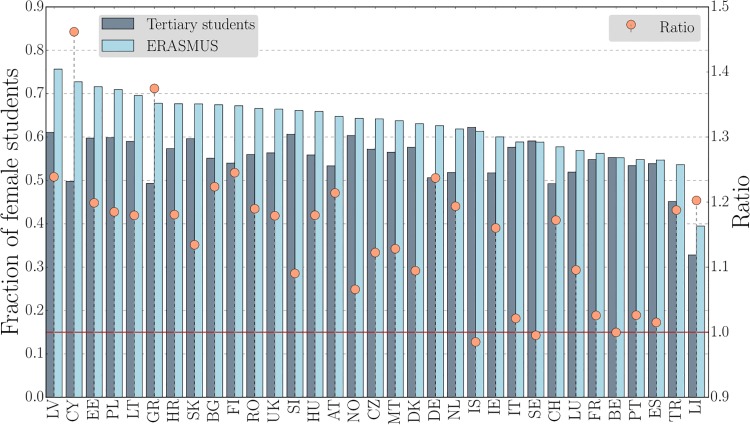
The over-representation of female students in the ERASMUS program is systematic across countries. Fraction of female students in the 2011–12 ERASMUS program (light blue) and in tertiary education in 2011 (dark blue) for the 33 participating countries. The orange circles are the ratios of these fractions. For Iceland, Italy, Sweden, France, Belgium, Portugal, and Spain, the fractions are similar. For all the other countries, female students are over-represented in the ERASMUS program in comparison to the tertiary student population.

The same gender asymmetry is observed across countries. Fig 3 contains the fraction of female students in the ERASMUS program, in tertiary education for all ERASMUS countries, and the ratio between them. Only seven out of 33 countries have a balanced fraction: Iceland, Italy, Sweden, France, Belgium, Portugal, and Spain. For all the other countries, female students are clearly over-represented in the ERASMUS program in comparison to the tertiary student population. The largest ratios are for Cyprus, Greece, Finland, Latvia, and Germany.

Let us now focus on the mobility between countries. For simplicity, we only consider ERASMUS exchanges between academic institutions, disregarding industrial placements (see *Materials and Methods*). In this way, one can keep track of institution names since they are uniquely defined, which is very important to geographically localize them. Table 1 contains the top five countries sending and receiving students in the ERASMUS program, ranked by the absolute number of ERASMUS outgoing and incoming students, respectively. Spain ranks first in both lists. In fact, approximately 30% of all ERASMUS students are either coming from or moving to Spain. The most popular university in the entire program is the University of Granada (Spain) that hosts roughly 2% of all ERASMUS students. If we normalize the number of outgoing students by the total number of tertiary students in the country, Spain ranks third (1.7% of tertiary students), being surpassed by Luxembourg (7.8% of tertiary students) and Liechtenstein (3.4%).

**Table 1:** Rank of sending and receiving countries. Top five countries with the highest number of outgoing (sending) and incoming (receiving) students in the ERASMUS program for academic exchange, i.e., study mobility. The fraction of female students is shown in brackets.

*Top sending*	*Top receiving*
Spain: 33634 (55%)	Spain: 30938 (67%)
Germany: 27106 (62%)	France: 22887 (69%)
France: 24250 (57%)	Germany: 20885 (59%)
Italy: 19757 (59%)	United Kingdom: 17697 (64%)
Poland: 11878 (71%)	Italy: 17028 (65%)

**Fig 4:**
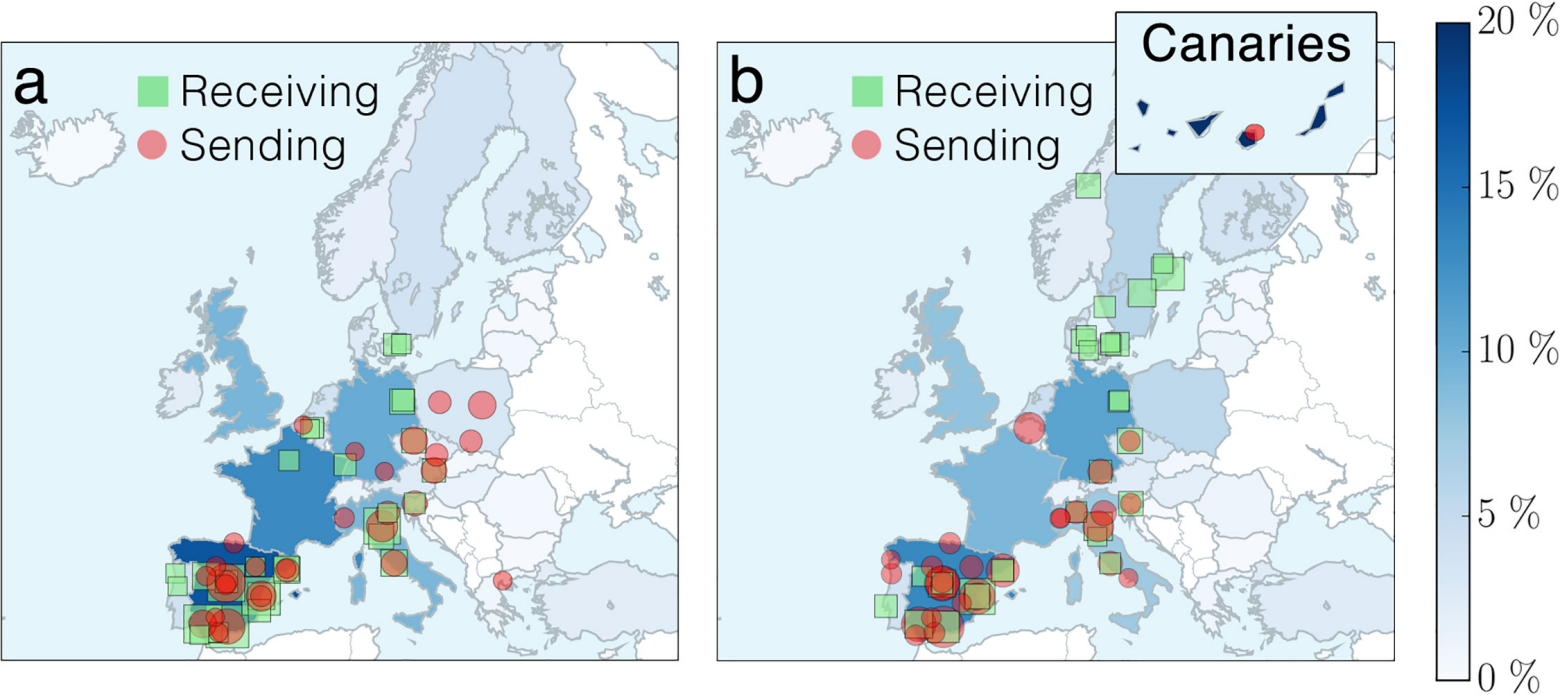
There is a gender asymmetry in the geographical distribution of involved institutions. Map of the top 30 academic institutions ranked by the number of outgoing and incoming (a) female and (b) male students. Red circles represent the sending institutions and green squares the receiving ones. The size of the symbols is proportional to the ratio of the number of ERASMUS students to the total number of ERASMUS students in the 30 academic institutions. The overall fraction of receiving students in each country is indicated by the intensity of the color of the country. The Scandinavian universities are much more attractive to male students than to female ones.

**Fig 5:**
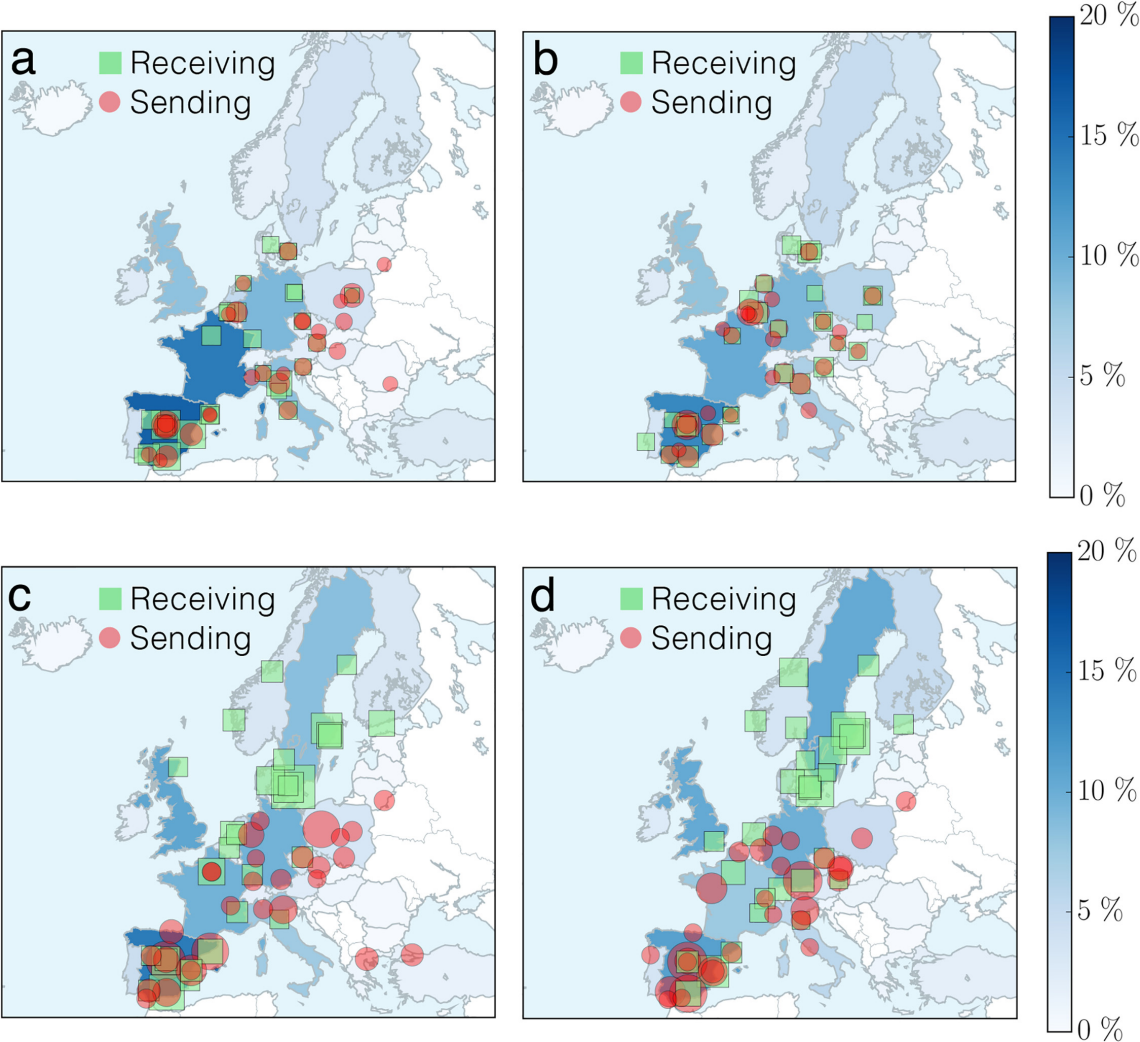
The geographical gender asymmetry is driven by subject area. Map of the top 30 academic institutions ranked by the number of outgoing and incoming (a,c) female and (b,d) male students, for (a,b) *social sciences, business and law* and (c,d) *natural sciences, mathematics and computing*. Red circles represent the sending institutions and green squares the receiving ones. The size of the symbols is proportional to the ratio of the number of ERASMUS students to the total number of ERASMUS students in the 30 academic institutions. The overall fraction of hosted students in each country is indicated by the intensity of the color of the country. When the ERASMUS participants are split into two groups (social sciences and sciences), the female and male mobility patterns are consistent.

From Table 1 one concludes that, for the top sending and receiving countries, female students are systematically over-represented. But, are there geographical regions that are preferred by female students more than by their male fellows? The maps in Fig 4 show the geographical distribution of the top 30 academic institutions ranked by the number of outgoing and incoming (a) female and (b) male students, revealing gender differences in the mobility pattern. Scandinavian universities are definitely more attractive to male students than to female ones. To understand this effect we analyze the mobility pattern of ERASMUS participants in the *social sciences, business and law* and *natural sciences, mathematics and computing*. For simplicity, we refer to them as *social science* and *science* groups, respectively. The social science group consists of 50496 female and 32011 male students. The science group is more balanced, with 7335 female and 8603 male students. Fig 5 shows the geographical distribution of the two different groups. Within the same subject area there are no significant gender differences. However, the patterns are significantly different between the two subject areas. This suggests that the observed gender differences in the geographical distribution of the top ranked institutions are rather driven by subject and not by gender.

## Discussion

The analysis of the mobility in the 2011–12 edition of the ERASMUS program reveals that female students tend to be over-represented, when compared to their participation in tertiary education. This over-representation is largely consistent across subject areas and countries. The study of the geographical distribution of home- and host-institutions also hints at a gender asymmetry, suggesting that Scandinavian institutions are more attractive to male students than to female ones. However, a more detailed analysis shows that the geographical asymmetry is driven by subject area and consistent with the distribution of gender ratios among subject areas.

In the present a study we aim to analyze the existing data without assuming any previous postulates. This study raises several social questions. What is the reason for this interesting gender gap in ERASMUS participation? Further studies are necessary. One direction for future work might be to investigate how social connections among participants affect their choice for the host-institution. For example, are friends applying for the same university to travel together? Could this be the mechanism underlying the geographical asymmetry? Also, empirical studies of the labor market suggest the opposite, namely that female workers are less mobile than their male partners [15], a gender gap that even increases for less-educated workers. The reason for this inversion is still elusive. It is also noteworthy that students from *sciences, mathematics and computing* go to Scandinavia more than they do for Spain and Italy together. This is in sharp contrast to the agglomerated data, which suggests that Spain and Italy are very popular countries.

## Supporting Information

S1 File ERASMUS data. Data set of ERASMUS participants.
(ZIP)
